# Evaluation of the Combined Effect of Artemisinin and Ferroptosis Inducer RSL3 against *Toxoplasma gondii*

**DOI:** 10.3390/ijms24010229

**Published:** 2022-12-23

**Authors:** Mao Huang, Xinru Cao, Yucong Jiang, Yuehong Shi, Yazhen Ma, Dandan Hu, Xingju Song

**Affiliations:** 1 College of Animal Science and Technology, Guangxi University, Nanning 530004, China; 2 Guangxi Zhuang Autonomous Region Engineering Research Center of Veterinary Biologics, Nanning 530004, China; 3 Guangxi Key Laboratory of Animal Reproduction, Breeding and Disease Control, Nanning 530004, China

**Keywords:** *Toxoplasma gondii*, dihydroartemisinin, ferroptosis inducer RSL3, mitochondrial membrane potential, ROS

## Abstract

*Toxoplasma gondii* is a widespread intracellular pathogen that infects humans and a variety of animals. Dihydroartemisinin (DHA), an effective anti-malarial drug, has potential anti-*T. gondii* activity that induces ferroptosis in tumor cells, but the mechanism by which it kills *T. gondii* is not fully understood. In this study, the mechanism of DHA inhibiting *T. gondii* growth and its possible drug combinations are described. DHA potently inhibited *T. gondii* with a half-maximal effective concentration (EC_50_) of 0.22 μM. DHA significantly increased the ROS level of parasites and decreased the mitochondrial membrane potential, which could be reversed by ferroptosis inhibitors (DFO). Moreover, the ferroptosis inducer RSL3 inhibited *T. gondii* with an EC_50_ of 0.75 μM. In addition, RSL3 enhanced the DHA-induced ROS level, and the combination of DHA and RSL3 significantly increased the anti-*Toxoplasma* effect as compared to DHA alone. In summary, we found that DHA-induced ROS accumulation in tachyzoites may be an important cause of *T. gondii* growth inhibition. Furthermore, we found that the combination of DHA and RSL3 may be an alternative to toxoplasmosis. These results will provide a new strategy for anti-*Toxoplasma* drug screening and clinical medication guidance.

## 1. Introduction

*T. gondii* is an obligate intracellular apicomplexan parasite that infects almost all warm-blooded animals, including humans and livestock, and causes severe toxoplasmosis in pregnant women and immunocompromised individuals. It is estimated that about one-third of the world’s population is infected with toxoplasmosis [1,2,3]. The current therapeutic strategy for human toxoplasmosis is a combination of sulphadiazine and pyrimethamine. However, this combination has little effect on chronic infections and has a certain failure rate [4,5,6,7]. Although other strategies are available, including the combination of pyrimethamine with clindamycin [8], atovaquone [9], clarithromycin [10], and either azithromycin or monotherapy with trimethoprim-sulfamethoxazole [11], no superiority over pyr-sulf has been found. Therefore, other drugs and combinations must be sought.

Artemisinin, a peroxide-containing sesquiterpene lactone isolated from the herb *Artemisia annua*, is an important drug for the treatment of malaria. Dihydroartemisinin (DHA), one of the derivatives of artemisinin, has a more stable and potent antimalarial effect than artemisinin [12]. Research on DHA in the apicomplexan parasite has focused on the mechanisms of clinical resistance. In *Plasmodium falciparum*, clinical resistance has been associated with multiple mechanisms, including genetic polymorphisms in PfKelch13 (K13), most notably K13C580Y [13,14]. DHA has also been found to have potential anti-*T. gondii* effects [15], and a role for the TCA and heme biosynthetic pathways and the mitochondrial protease DegP2 in the clinical resistance of *T. gondii* to DHA has been reported [16]. However, the exact mechanism by which DHA kills *T. gondii* is still unclear.

Ferroptosis is a novel type of cell death characterized by iron accumulation and ROS-dependent generation of lethal amounts of lipid hydroperoxides [17]. DHA has been shown to exert a strong inhibitory effect on a variety of cancer cell activities through iron death, including head and neck carcinoma cells, glioma cells, leukemia cells, and pancreatic ductal adenocarcinoma [18,19,20,21]. Therefore, an increasing number of ferroptosis inducers such as erastin [22], Ras-selective lethal small molecule 3 (RSL3), sorafenib, sulfasalazine, statins, and artemisinin has been considered as potential cancer drugs [22,23,24,25]. For the apicomplexan parasite, previous studies have shown that low doses of DHA can induce ferroptosis in *P. falciparum*, causing the accumulation of excessive hydroxyl radicals in the parasites and ultimately leading to parasite death [26]. However, little is known about the involvement of ferroptosis in *T. gondii* and the effect of the combination of DHA and ferroptosis inducers.

Considering the anti-*T. gondii* effect of DHA and its property of inducing ferroptosis in cells, we were curious whether DHA could also kill *T. gondii* by inducing ferroptosis in parasites. In this study, the molecular mechanism of DHA inhibiting *T. gondii* growth was revealed from the perspective of oxidative stress. Furthermore, we evaluated the efficacy of DHA combined with ferroptosis inducers against *T. gondii* in vitro. This study may provide some new thoughts for anti-*T. gondii* drug development.

## 2. Results

### 2.1. DHA Treatment Causes Growth Inhibition and Excessive ROS Accumulation in T. gondii Tachyzoites

**Figure 1:**
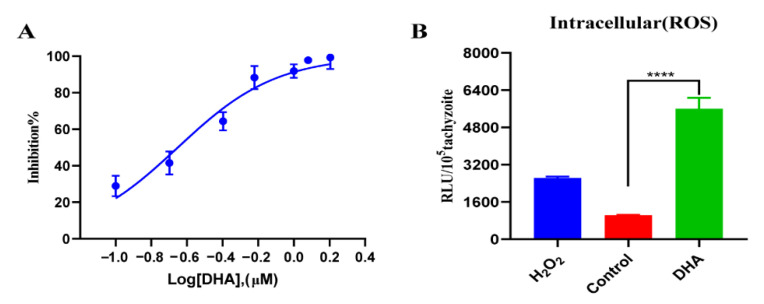
DHA treatment causing growth inhibition and excessive ROS accumulation in *T. gondii*. (**A**) Antiparasitic effect of DHA. Vero cells were infected with 1 × 10^5^ TgRH-Luc tachyzoites and treated with different concentrations of DHA for 24 h. Percentage inhibition was calculated based on the relative RLU in each treatment group and DMSO control. (**B**) DCFDA determination of ROS levels of parasites under DHA treatment. Vero cells were infected with 4 × 10^5^ TgRH-Luc tachyzoites for 12 h and then treated with DHA (0.88 μM) for 24 h. Positive and solvent controls were treated with H_2_O_2_ (200 μM) or DMSO for 4 h, respectively. Results were based on RLU per 10^5^ tachyzoites in each group. The stability and effectiveness of this method were validated in three independent experiments. Statistical analysis was performed using Student’s *t*-tests, and **** indicates *p* < 0.0001.

To evaluate the anti-parasitic effect of DHA, TgRH-Luc tachyzoites were treated with a serial dilution of DHA, and growth inhibition was determined after 24 h based on relative RLU. The results showed a dose-dependent inhibitory effect of DHA on *T. gondii* tachyzoites with an EC_50_ of 0.22 μM (95% confidence interval [CI], 0.1 to 1.2 µM) (Figure 1A).

ROS-dependent generation has been suggested as a possible signal for ferroptosis. To explore whether DHA affects the ROS level of *T. gondii*, we measured the parasite’s ROS levels after DHA treatment. DMSO or 200 μM H_2_O_2_-treated tachyzoites were used as controls, respectively. Our results showed that DHA treatment resulted in a five-fold ROS accumulation compared to DMSO treatment (Figure 1B).

### 2.2. DHA Causes a Dose-Dependent Increase in ROS Levels in Tachyzoites

**Figure 2:**
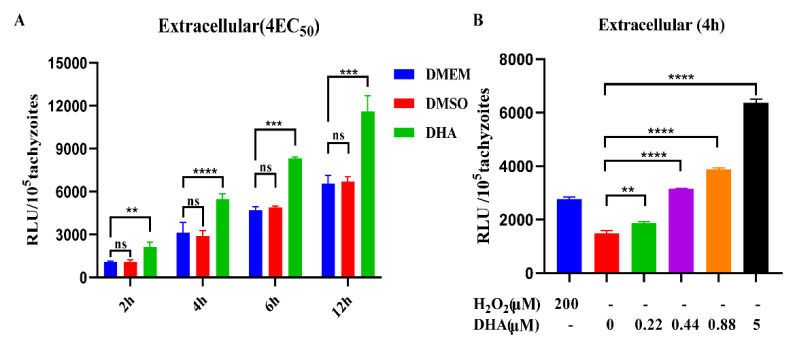
DHA-induced dose-dependent ROS production in tachyzoites. (**A**) ROS levels of parasites after different times of DHA treatment. Fresh extracellular tachyzoites were collected and incubated with 0.88 µM DHA at 37 °C for 2, 4, 6, and 12 h, respectively. Then, RLUs of tachyzoites in each sample were detected using the DCFDA probe. DMSO was treated as a control. (**B**) Effect of different DHA concentrations on ROS levels in extracellular tachyzoites. Fresh extracellular tachyzoites were incubated with 0 to 5 µM DHA or DMSO at 37 °C for 4 h, and then RLUs were detected. H_2_O_2_ (200 µM) was used as a positive control. Statistical analysis was performed using Student’s *t*-tests; ns, not significant; ** *p* < 0.05, *** *p* < 0.01 and **** *p* < 0.001. Three independent experiments were performed.

To further understand the characteristics of DHA-induced ROS production in *T. gondii*, we evaluated the timing and dose patterns of ROS production in DHA-treated tachyzoites. The results showed that ROS levels in extracellular tachyzoites increased with time in the absence of DHA. However, it is worth noting that during the same time period, ROS levels in DHA-treated tachyzoites were significantly higher than those in the untreated group (*p* < 0.001) (Figure 2A). Additionally, tachyzoites were treated with different concentrations of DHA (0.22, 0.44, 0.88, 5 µM) for 4 h, and a dose-dependent increase in ROS levels was found in tachyzoites compared to DMSO treatment (Figure 2B).

### 2.3. DHA Treatment Reduces Mitochondrial Membrane Potential

**Figure 3:**
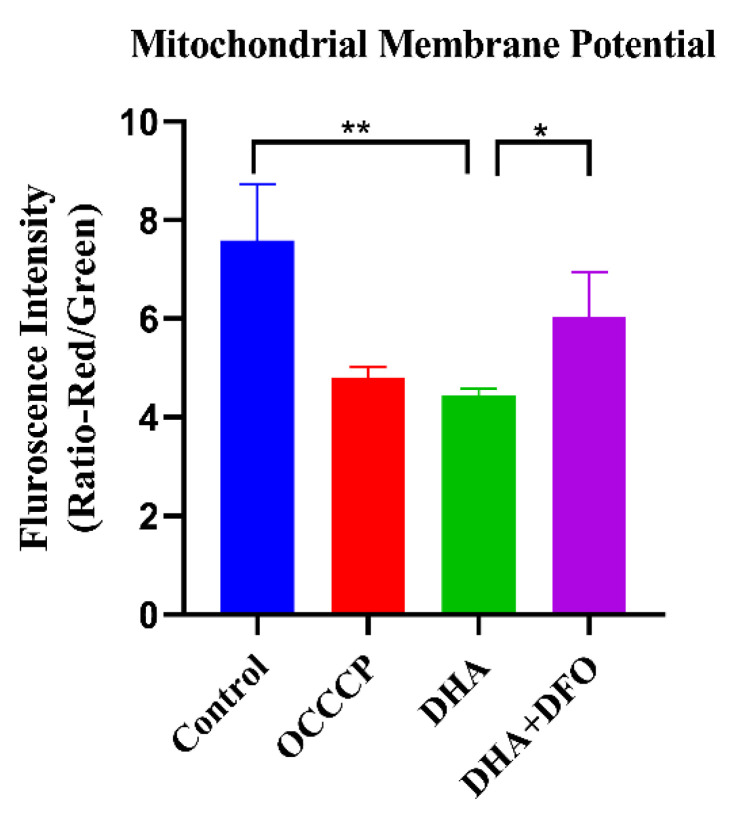
DHA treatment reduces mitochondrial membrane potential. Freshly released tachyzoites were treated with 4 × EC50 (0.88 μM) DHA or DHA + 80 μM DFO at 37 °C for 4 h. The FI signals of the mitochondrial membrane potential fluorescent probe JC-10 were then detected. Quantification of the red/green fluorescence intensity ratio was calculated by comparing the red and green channels per 10^5^ tachyzoites. OCCCP was treated as a positive control. Student’s *t*-tests were used to determine statistical significance (** *p* < 0.01, and * *p* < 0.05).

DHA treatment has been reported to be associated with mitochondrial damage in *T. gondii*. Ferroptosis causes mitochondrial depolarization, which directly leads to a reduction in mitochondrial membrane potential. Therefore, to evaluate the effect of DHA on mitochondria, we measured the mitochondrial membrane potential using JC-10. The mitochondrial membrane potential was significantly reduced after DHA treatment compared to the DMSO control. Moreover, we found that the DHA-induced decrease in mitochondrial membrane potential could be rescued by 80 μM DFO (Figure 3).

### 2.4. DHA Treatment Decreases the Transcription Levels of Important Antioxidant Genes in T. gondii

**Figure 4:**
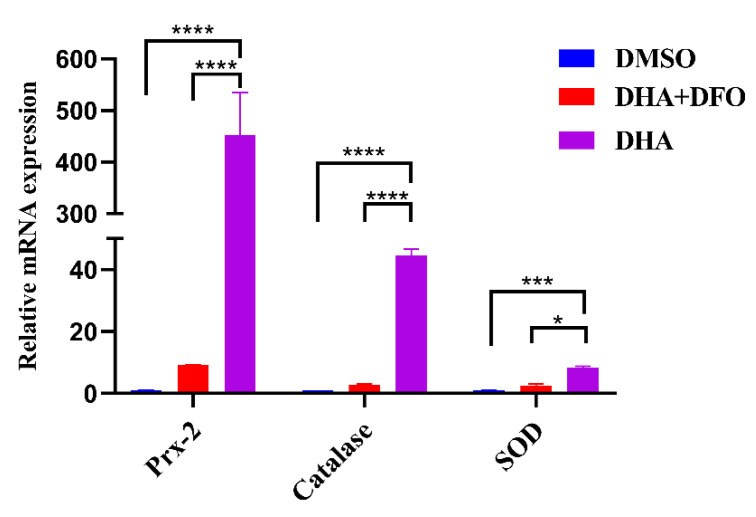
qPCR analysis of antioxidant genes. Equal amounts of fresh tachyzoites were inoculated into Vero cells and grown for 12 h. Tachyzoites were collected for qRT-PCR analysis after 24 h of treatment with 0.88 μM DHA, DHA + DFO, or DMSO. Experiments were performed in three independent experiments. Student’s *t*-tests were used for this analysis (* *p* < 0.05, *** *p* < 0.001, and **** *p* < 0.0001).

*T. gondii* expresses a variety of antioxidant enzymes, which can remove excess exogenous or endogenous ROS. However, DHA treatment resulted in the accumulation of ROS in tachyzoites. Therefore, we further tested the transcriptional levels of important antioxidant enzymes (PRX-2, Catalase, and SOD) in tachyzoites after DHA treatment by qPCR. We found that the transcriptional levels of PRX-2 (452-fold, *p* < 0.0001), Catalase (44-fold, *p* < 0.0001), and SOD (8-fold, *p* < 0.001) were all dramatically increased after DHA treatment (Figure 4). Notably, the increased mRNA levels of antioxidant genes were significantly down-regulated by the ferroptosis inhibitor DFO (Figure 4). Interestingly, we found that *T. gondii* PRX2 is homologous to the key cellular ferroptosis marker gene GPX4 by homology alignment, which may indicate that DHA is related to *T. gondii* ferroptosis, but it still needs further validations.

### 2.5. DHA and Ferroptosis Inducers Simultaneously Increase ROS in T. gondii

**Figure 5:**
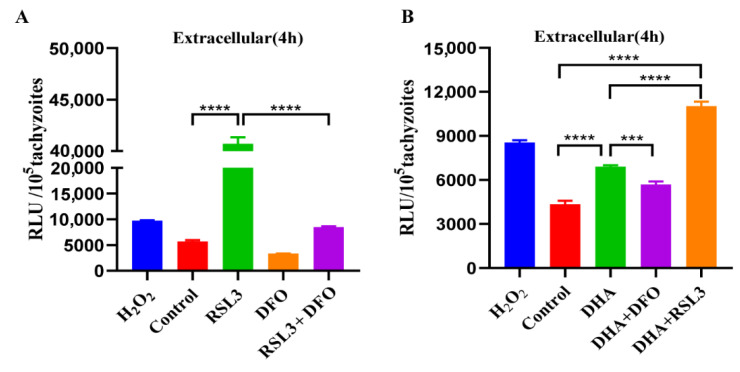
Effect of the ferroptosis inducer and inhibitor on ROS levels of *T. gondii.* (**A**) Effects of ferroptosis inducer RSL3 and inhibitor DFO on ROS of *T. gondii*. Fresh tachyzoites were incubated at 37 °C and exposed to DMSO, 3 μM RSL3 (4 × EC_50_), or 3 μM RSL3 + 80 μM DFO for 4 h. Then, the fluorescence intensity of ROS was detected using DCFDA. (**B**) The effect of DHA combined with the ferroptosis inducer or inhibitor on the ROS level of *T. gondii*. The mean fluorescence intensity of ROS treated with compounds DHA, DHA + DFO, and DHA + RSL3 was detected. Experiments were performed in triplicate. Statistical analysis used Student’s *t*-tests (*** *p* < 0.001, and **** *p* < 0.0001).

The generation of ROS and up-regulation of the PRX2 gene after DHA treatment have been considered as possible signs of ferroptosis. We further validated this using a ferroptosis inducer (RSL3) and an inhibitor (DFO). The ferroptosis inducer RSL3 significantly increased ROS in *T. gondii*, and it could be significantly blocked by DFO (*p* < 0.0001) (Figure 5A). In addition, we found that RSL3 combined with DHA treatment significantly increased ROS levels compared to the DMSO control group (*p* < 0.0001) or the DHA-treated group (*p* < 0.0001) (Figure 5B). On the contrary, the ROS level of DHA combined with DFO treatment was significantly reduced compared to the DHA-treated group (*p* < 0.001) (Figure 5B). These results indicated that the ferroptosis inducer RSL3 could significantly enhance the DHA-induced ROS level, suggesting that RSL3 may be combined with DHA for the treatment of toxoplasmosis.

### 2.6. The Ferroptosis Inducer RSL3 Has a Potent Inhibitory Effect on Intracellular and Extracellular Tachyzoites In Vitro

**Figure 6:**
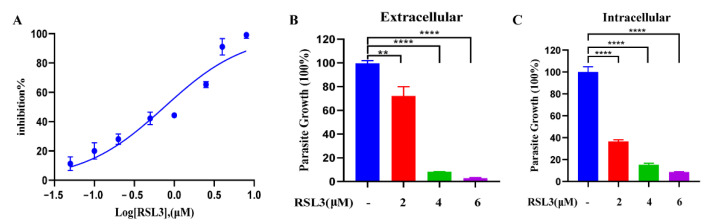
Evaluation of the anti-*Toxoplasma* effect of RSL3. (**A**) RSL3 can potently inhibit the growth of *T. gondii*. Vero cells were infected with 1 × 10^5^ tachyzoites and treated with different concentrations of RSL3 for 24 h. The inhibition rate was then calculated based on the relative RLU in each treatment group and DMSO control. (**B**) The effect of RSL3 on extracellular parasites. Fresh extracellular tachyzoites (1 × 10^5^) were pretreated with 0, 2, 4, and 6 µM RSL3 for 4 h and then inoculated into cells and cultured in drug-free medium for 20 h. DMSO was used as a solvent control. The inhibition rate was calculated using the RLUs of the RSL3- and DMSO-treated groups. (**C**) The effect of RSL3 on intracellular parasites. Tachyzoites were allowed to infect the cells for 4 h, and then non-invasive tachyzoites were removed, and the remaining parasites were cultured for 20 h in medium containing 0, 2, 4, and 6 µM RSL3. Statistical analysis used Student’s *t*-tests (** *p* < 0.05, and **** *p* < 0.001). Experiments were performed in triplicate.

Excessive amounts of ROS in *T. gondii* could lead to parasite death. Thus, compounds that induce ROS production may have the potential to treat toxoplasmosis. Our above experiments showed that RSL3 could induce excessive ROS accumulation in parasites. To further investigate the anti-*T. gondii* effect of RSL3, the growth inhibition assay was performed in vitro. RSL3 showed a dose-dependent inhibitory effect on *T. gondii* tachyzoites with an EC_50_ of 0.75 µM (95% confidence interval [CI], 0.05 to 6 µM) (Figure 6A). We also examined the inhibitory effect of different concentrations of RSL3 on intracellular and extracellular tachyzoites, respectively. The results showed that RSL3 significantly inhibited the growth of extracellular *T. gondii* in a dose-dependent manner (Figure 6B). For the intracellular assay, RSL3 also exhibited a potent inhibitory effect (Figure 6C). These results suggest that RSL3 has a potent inhibitory effect on intracellular and extracellular tachyzoites in a dose-dependent manner.

### 2.7. Antiparasitic Effects of DHA with Ferroptosis Inducers and Inhibitors

**Figure 7:**
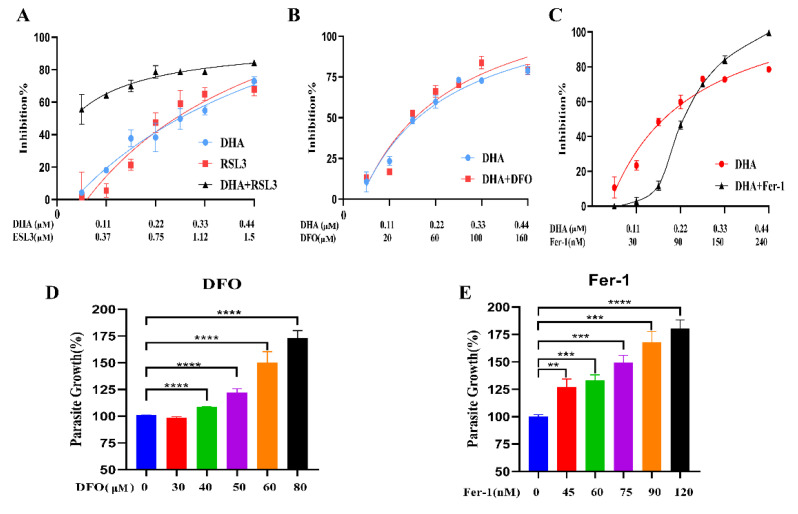
Inhibition of *T. gondii* growth by a combination of DHA and ferroptosis inducers or inhibitors. (**A**) Dose–effect curves of DHA, RSL3 alone, and DHA + RSL3. (**B**) Dose–effect curves of DHA alone and DHA in combination with DFO. (**C**) Dose–effect curves of DHA alone and DHA in combination with Fer-1. (**D**,**E**) Parasite growth rates with DFO or Fer-1 at different doses. Statistical analysis used Student’s *t*-tests (** *p* < 0.05, *** *p* < 0.001, and **** *p* < 0.0001). Each combination was set up in triplicate wells, and the experiments were independently performed at least three times.

Since the ferroptosis inducer RSL3 significantly enhanced DHA-induced ROS levels, we hypothesized that the combination of RSL3 and DHA might have a better effect on *T. gondii*. To verify our hypothesis, we measured the growth inhibition of tachyzoites by the combination of DHA and other ferroptosis inducers (RSL3) or inhibitors (DFO and Fer-1). Our results showed that the combination of RSL3 with DHA significantly (*p* < 0.001) increased parasite growth inhibition compared to treatment with DHA or RSL3 alone (Figure 7A). Moreover, the combinations DHA + DFO or DHA + Fer-1 did not show significant (*p* > 0.05) changes in parasite growth compared to DHA treatment alone (Figure 7B,C). In addition, we were surprised to find that both DFO and Fer-1 significantly (*p* < 0.001) promoted the growth of parasites and presented dose-dependent growth (Figure 7D,E). Notably, when the parasite was pretreated with 0.22 µM DHA, its growth ability was significantly restored with increasing DFO concentrations (Figure S1).

### 2.8. RSL3 and DHA Is Not Toxic to Host Cells at Antiparasitic Concentration

**Figure 8:**
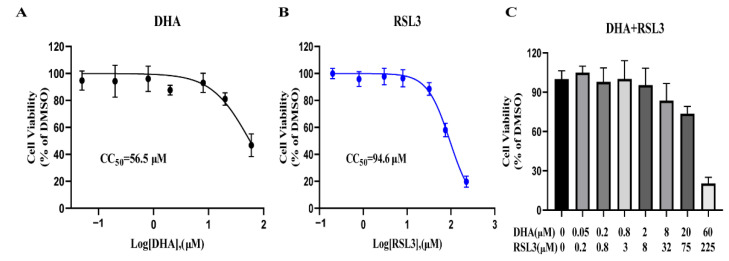
Cytotoxicity of RSL3 and DHA on Vero cells. The cytotoxicity of 0.05–60 µM DHA (**A**), 0.2–225 µM RSL3 (**B**), and 0.2–225 µM RSL3 plus 0.05–60 µM DHA (**C**) on Vero cells was measured using CCK-8 reagent, respectively. The concentration combination ranged from 0.25 to 300 × EC_50_ DHA and RSL3. DMSO was set as a control. Cell viability rate = OD RSL3/OD DMSO. This experiment was performed in triplicate.

To assess the cytotoxic effects of these drugs alone (DHA, RSL3, DFO, Fer-1) or their combinations (DHA with RSL3, DHA with DFO, and DHA with Fer-1) on host cells, serial diluted drugs were added to host cells, and cell viability was determined using CCK-8 reagent. The results showed that the 50% cytotoxicity concentrations (CC_50_) of RSL3 and DHA were 94.6 μM and 56.5 μM, respectively (Figure 8A,B). The CC_50_ values of RSL3 and DHA for Vero cells were 256- and 126-fold higher than the EC_50_ values against *T. gondii*, which demonstrated that RSL3 and DHA have high therapeutic indices. In addition, the combination of DHA with RSL3 was not toxic to cells under the therapeutic concentration (Figure 8C). DFO and Fer-1 had a growth promotion effect on the Vero cells, and these combinations (DHA + DFO and DHA + Fer-1) also showed no toxic to cells under the therapeutic concentration (Figure S2).

## 3. Discussion

*T. gondii* infects almost all warm-blooded animals and humans. It is fatal or may cause serious problems in fetuses and immunocompromised patients. However, there is currently no approved and commercially available vaccine against toxoplasmosis [27]. Clinical treatment of toxoplasmosis relies on chemical drugs, but there are still serious side effects and limitations [4,5,6,7,28]. Therefore, there is an urgent need to develop novel components or combinations that can effectively and safely combat *T. gondii*. DHA is a derivative of artemisinin, which has been shown to have a potent therapeutic effect against malaria. DHA has also been found to have anti-*Toxoplasma* effects, particularly by reducing cysts in mice [15,29]. Our study shows that DHA has a dose-dependent inhibitory effect on *T. gondii.* Furthermore, DHA treatment increased ROS accumulation in a dose-dependent manner, which suggested that ROS accumulation in tachyzoites caused by DHA may be one of the important reasons for inhibiting parasite growth.

Previous studies have shown that DHA causes ferroptosis in many cancer cells [30,31] and in *P. falciparum* [26]. Ferroptosis was first demonstrated by Dixon in 2012 [32] as a regulated form of cell death that is iron- and ROS-dependent and has attracted much attention in many territories, especially in oncology. Thus, ROS-dependent generation is considered to be an important signal for ferroptosis [33]. In this study, we demonstrate that DHA treatment can lead to an increase in ROS in tachyzoites and can be rescued by the ferroptosis inhibitor DFO. The ferroptosis inducer RSL3 can directly bind to GPx4 and inhibit its activity, leading to the intracellular accumulation of lipid peroxides and cell ferroptosis [34]. In our study, the ferroptosis inducer RSL3 significantly increased the level of DHA-induced ROS. This suggests that the anti-*Toxoplasma* mechanism of DHA is closely related to ferroptosis. Furthermore, depolarized mitochondrial membrane potential has been reported as a new characteristic of ferroptosis [35,36]. Mitochondrial depolarization leads to a decrease in mitochondrial membrane potential. In our study, the mitochondrial membrane potential decreased after DHA treatment, suggesting that DHA treatment may be associated with ferroptosis.

Peroxiredoxins, catalase, and superoxide dismutase form an antioxidant network in *T. gondii*, scavenging free hydroxyl radicals, superoxide and hydrogen peroxide [37]. Glutathione peroxidase 4 (GPX4) is an antioxidant enzyme that has been shown to be critical for ferroptosis in cells [38] and is highly homologous to the novel peroxiredoxin (Prx) family of proteins [37]. Prxs may provide defense against oxidative damage and appear to be involved in the control of H_2_O_2_ concentration [39]. Catalase, a potent H_2_O_2_-detoxifying enzyme, is unexpectedly absent in some members of the Kinetoplastida and Apicomplexa, but it is present in *T. gondii* [40]. SODs comprise a family of ubiquitous enzymes that provide essential protection to biological systems against uncontrolled reactions with oxygen- and nitrogen-based radical species [41]. Previous studies have shown that several compounds can lead to ROS generation, which disrupts mitochondrial antioxidant enzymes such as PRX, SOD, and catalase [42,43,44,45,46]. In this study, the transcriptional levels of Prx-2, catalase, and SOD were dramatically increased in response to DHA treatment. Interestingly, we found that *T. gondii* PRX2 was homologous to the cellular ferroptosis marker gene GPX4 by sequence alignment. Furthermore, the transcriptional level of Prx-2 showed a significant decrease in the combination of DHA and DFO compared to DHA-treated parasites. These results further suggest that the antiparasitic mechanism of DHA may be largely related to iron death.

Elevated ROS levels have been shown to contribute to the antimicrobial activities of certain antibiotics, especially quinolone-based drugs [47,48]. Secondary damage occurs upon ROS accumulation, including disruption of mitochondrial DNA, nucleic acid, lipid, and protein synthesis [47,48,49,50]. *T. gondii* is highly sensitive to ROS, and small changes may lead to disruption of oxidative homeostasis and ultimately to parasite death [51,52], and excessive ROS can inhibit or even kill *T. gondii*. Since the ferroptosis inducer RSL3 significantly enhances the DHA-induced ROS level, we evaluated the anti-*T. gondii* efficacy of RSL3 in combination with DHA. The results showed the RSL3 significantly enhanced the anti-*Toxoplasma* effect of DHA. In contrast, the anti-*T. gondii* efficacy of the combination of DHA with ferroptosis inhibitor was not significantly compared with DHA treatment alone. Notably, when the parasites were pretreated with 0.22 µM DHA, the growth ability of the parasites was significantly restored with the increase of DFO concentration. These results indicated that the anti-*T. gondii* efficacy of DHA may be associated with ferroptosis. In addition, the ferroptosis inhibitor DFO and Fer-1 can promote the growth of *T. gondii*, which is similar to the study in malaria where Liproxstatin-1, another ferroptosis inhibitor, could also promote parasite growth [26]. However, whether DHA may damage the *Toxoplasma* membrane through polyunsaturated fatty acid attack remains unknown and requires further study.

## 4. Materials and Methods

### 4.1. Parasites and Cell Culture

Green monkey kidney cells (Vero) were purchased from ATCC (Manassas, VA, USA) and cultured in Dulbecco’s modified Eagle’s medium (DMEM) supplemented with 10% fetal bovine serum. The type I RH strain of *T. gondii* expressing luciferase (TgRH-Luc) at the UPRT site was used and maintained in Vero cells in DMEM supplemented with 2% fetal bovine serum at 37 °C and 5% CO_2_.

### 4.2. Compounds

The following drugs were tested for in vitro assay: DHA (Aladdin Holdings Group Co., Ltd., Shanghai, China), RSL3 (MedChemExpress, Shanghai, China), Ferrostatin-1 (MedChemExpress, Shanghai, China), and DFO (MedChemExpress, Shanghai, China). All compounds were dissolved in 100% DMSO and sterilized through 0.22 µm filter membranes.

### 4.3. Growth Inhibition Assay

The in vitro anti-*Toxoplasma* activity of all drugs was investigated using TgRH-Luc tachyzoites. Vero cells were seeded onto 96-well cell plates and cultured at 37 °C under 5% CO_2_ until 100% confluence was achieved. Cells were then infected with 2 × 10^5^ of TgRH-Luc per well. After 24 h, DHA (0.05 to 1.6 μM), RSL3 (0.05 to 2.5 μM), or a combination of DHA and ferroptosis inducer/inhibitor were serially diluted into each parasite-infected well. Equal amounts of DMSO were used as controls. The relative luminescence units (RLU) of the parasites were detected after 24 h by a fluorescence microplate reader (Tecan, Infinite M200 PRO, Männedorf, Switzerland) using the Bright-Lumi™ II Firefly Luciferase Assay Kit (Beyotime Biotech, Shanghai, China). Percent growth inhibition was calculated as follows: inhibition rate = [(RLU DMSO − RLU treatment)/RLU DMSO] × 100%. Samples were run in triplicate, and three independent assays were performed. Data were expressed as means ± standard deviation (SD). Half-maximal effective concentrations (EC_50_) of compounds and 95% confidence intervals (CI) were extrapolated using the log (inhibitor) versus response-variable slope (four-parameter) regression equation in GraphPad Prism 8 (GraphPad, La Jolla, CA, USA).

### 4.4. ROS Assay

Extracellular TgRH-Luc tachyzoites were treated with DHA at different concentrations (0.22 to 5 μM) and times (2 to 12 h) at 37 °C. Then, 10 μM 2′,7′-dichlorofluorescein diacetate (DCFDA) (Sigma, St. Louis, MA, USA) was labeled in the tachyzoites for 20 min in the dark at 37 °C. After three washes, the tachyzoites were resuspended with DMEM, and the RLU signals (wavelength of Ex = 488 nm/Em = 525 nm) of DCFDA were obtained using a fluorescence microplate reader (Tecan, Infinite M200 PRO, Männedorf, Switzerland). The final value of ROS for each group was expressed as RLU/parasite. DMSO and 200 μM H_2_O_2_-treated tachyzoites were used as controls, respectively.

### 4.5. Mitochondrial Membrane Potential (Δψ) Assay

First, the freshly released tachyzoites were treated with 4 × EC_50_ (0.88 μM) DHA and incubated at 37 °C for 4 h. Then, they were resuspended with 0.5 mL of Mitochondrial Membrane Potential Fluorescent probe (JC-10) (Biosharp, Shanghai, China) and subsequently incubated in the dark at 37 °C for 20 min. The tachyzoites were then washed three times in the dark using JC-10 staining buffer and resuspended in JC-10 staining buffer. OCCCP was used as a positive control. The number of parasites in each group was counted to calculate Δψ in each group. Finally, the FI signals of JC-10 (JC-10 Monomer wavelength of Ex = 490 nm/Em = 530 nm; JC-10 Polymer wavelength of Ex = 525 nm/Em = 590 nm) were detected using a fluorescence microplate reader (Tecan, Infinite M200 PRO, Männedorf, Switzerland). The final Δψ for each group was calculated as the RLU divided by the number of tachyzoites in each group.

### 4.6. Quantitative Real-Time (qPCR) Analysis of Antioxidant Genes

RNA was extracted from fresh tachyzoites treated with DHA or DHA + DFO for 24 h by RNA extraction kit (Aidlab biotechnologies, Beijing, China) and then reverse transcribed to cDNA using StarScript III All-in-one RT Mix with gDNA Remover (GenStar, Fuzhou, China). Transcription levels of antioxidant genes, including peroxiredoxin-2 (Prx-2), catalase, and superoxide dismutase (SOD), were quantitated by Real-time qPCR using 2× RealStar Green Fast Mixture (GenStar, China). Data were acquired using LightCycler^®^ 96 (Roche, Basel, Switzerland) and normalized to TgActin expression levels. The sequences of the primers used are listed in Supplementary Table S1.

### 4.7. Cytotoxicity Test

The cytotoxicity of these drugs used alone (DHA, RSL3, DFO, Fer-1) or their combinations (DHA with RSL3, DHA with DFO, and DHA with Fer-1) were evaluated in Vero cells using CCK-8 reagent (Beyotime, Shanghai, China). Briefly, Vero cells (5000 cells/well) were cultured in 96-well-plates at 37 °C and 5% CO_2_ for 24 h. Serial diluted drugs were added to the cultures separately and incubated for additional 24 h. Then, cell viability was determined using CCK-8 reagent according to the manufacturer’s instructions. Absorbance was measured at 450 nm using a Microplate Absorbance Reader (BioRad, Hercules, CA, USA). DMSO was set as a control. Cell viability was calculated as OD_RSL3_/OD_DMSO_. The cytotoxicity experiment was performed in triplicate.

### 4.8. Statistical Analysis

Graphs and statistical analysis were performed using Graph Pad Prism (San Diego, CA, USA). Graphs represent means and error bars represent standard errors of the means. These data were analyzed using two-tailed Student’s *t*-tests; *p*-values are indicated by asterisks in the figures as follows: * *p* < 0.05, ** *p* < 0.01, *** *p* < 0.001, and **** *p* < 0.0001. We considered all *p*-values < 0.05 to be significant.

## 5. Conclusions

This study showed that DHA significantly increased the ROS level of *T. gondii* and decreased the mitochondrial membrane potential. The elevated ROS level could be reversed by the ferroptosis inhibitor DFO. The ferroptosis inducer RSL3 potently inhibited the growth of *T. gondii* and enhanced the DHA-induced ROS level. The combination of DHA and RSL3 significantly increased the anti-*Toxoplasma* effect compared to DHA alone. These data may provide a new strategy for anti-*Toxoplasma* drug development.

## Data Availability

All datasets generated for this study are included in the manuscript/supplementary files.

## Supplementary Materials

The supporting information can be downloaded at: https://www.mdpi.com/article/10.3390/ijms24010229/s1.
