# Supplementary material for: Evaluation of the Combined Effect of Artemisinin and Ferroptosis Inducer RSL3 against Toxoplasma gondii
Source: Int J Mol Sci. 2022 Dec 23;24(1):229. doi: 10.3390/ijms24010229 (PMC9820390; doi:10.3390/ijms24010229)
Supplement: Supplementary file 1 [file ijms-24-00229-s001.zip › ijms-2038054-supplementary.pdf]

Supplementary materials:

Table S1. The sequence of qPCR primers used in this study.

Gene Name	Gene number	Forward Primer (5'-3')	Reverse Primer (5'-3')
Prx-2	TGARI_266130	AATCTCACAGGGCACTTTGGGAAG	CTCTACTAGGCGAAGCGGGTTTG
SOD	TGARI_316310	CAATGTCTGGTGGTGGTGGAGAAC	ATGCCCAACCTGAGCCAAAGTG
Catalase	TGARI_232250	CAGGGATTGAACCGAGCGAAGATC	GCCAAGTCTGTGCCGATGAGTG

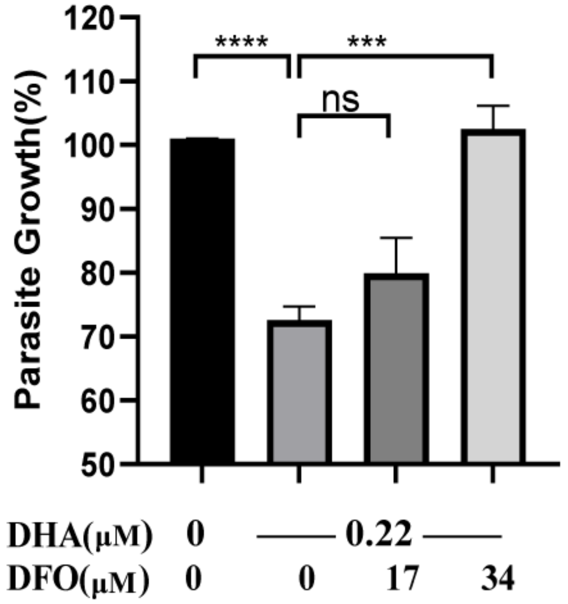

Figure S1. Ferroptosis inhibitor DFO sresces the anti-parasitic effect of DHA. Tachyzoites were pretreated of with 0.22 μM DHA for 2h, then DHA was removed and 0 - 34 μM DFO was added. Subsequently, the fluorescence value of TgRH Luc was measured after 22h of exposure. Statistical analysis used Student's t test (*, P < 0.05, ***, P < 0.001, ****, and P < 0. 0001). Experiments were performed in triplicate.

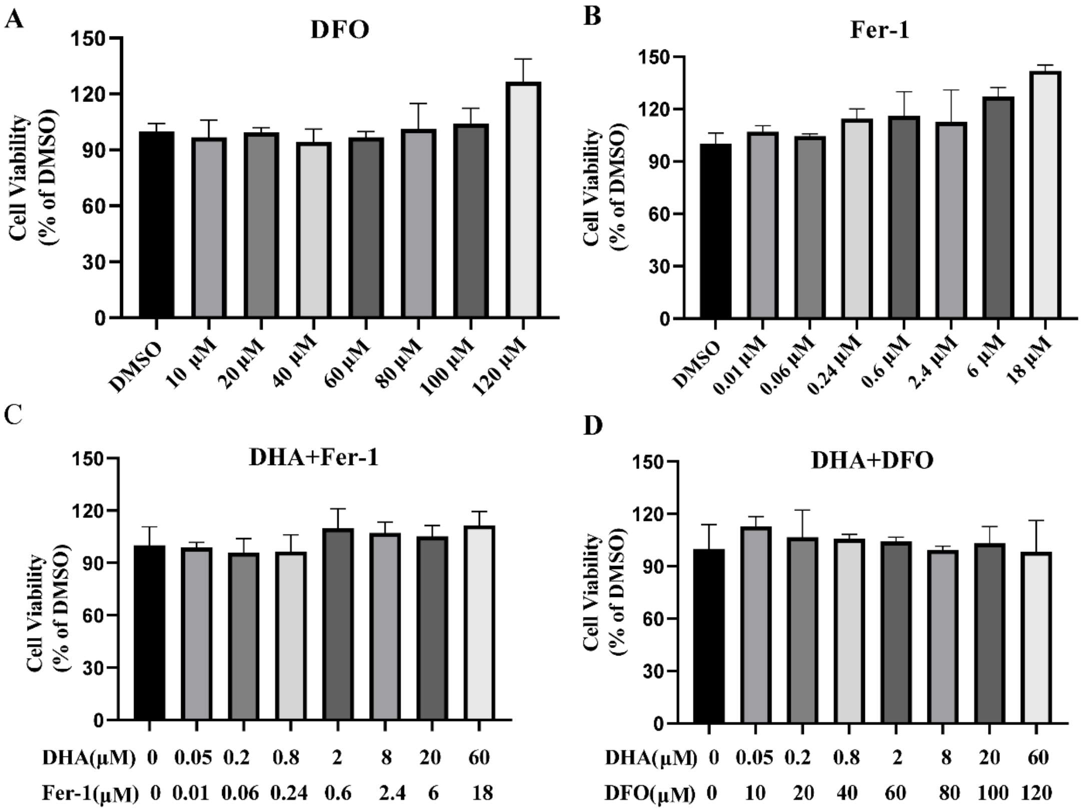

Figure S2. Cytotoxicity of DFO and Fer-1 on Vero cells. Cytotoxicity of 10-120 μM DFO (A), 0.01-18 μM Fer-1(B), 0.01-18 μM Fer-1 supplemented with 0.05-60 μM DHA (C), and 10-120 μM DFO supplemented with 0.05-60 μM DHA (D) on Vero cells was measured using CCK-8 reagent respectively. DMSO was set as a control. Cell Viability rate = OD RSL3/OD DMSO. This experiment was performed in triplicate.
